# Can artificial systems become conscious?—Recursive embodied closure as an integrative framework

**DOI:** 10.3389/fpsyg.2026.1950309

**Published:** 2026-09-30

**Authors:** Achim Schilling, Patrick Krauss

**Affiliations:** 1 BCI and NeuroAI Group, Neuromodulation and Neuroprosthetics, University Hospital Mannheim, University Heidelberg, Mannheim, Germany; 2 BG Clinic Ludwigshafen, Ludwigshafen, Germany; 3 Physics and Cognition Group, Neuromodulation and Neuroprosthetics, University Hospital Mannheim, University Heidelberg, Mannheim, Germany; 4 Cognitive Computational Neuroscience Group, Pattern Recognition Lab, University Erlangen-Nürnberg, Erlangen, Germany

**Keywords:** artificial intelligence, consciousness, embodiment, machine consciousness, predictive processing, self models, theoretical neuroscience, world models

## Abstract

Recent advances in artificial intelligence, connectomics, and computational neuroscience increasingly raise the question of whether artificial systems—or even pure computer simulations—could become conscious. Contemporary theories emphasize embodiment, predictive processing, homeostatic regulation, and continuous interaction between cognition, body, and environment rather than isolated computation. We propose recursive embodied closure as an integrative framework linking embodied, predictive, functionalist, and dynamical approaches to consciousness. Rather than replacing existing theories, the framework identifies a shared organizational principle: the continuous recursive coupling of cognition, bodily regulation, action, and environmental feedback. We argue that consciousness may, in principle, emerge in artificial systems, including computational simulations, for three reasons. First, consciousness may depend on recursively closed loops linking cognition, body, and environment, and such loops could also be realized in robotic, neuromorphic, or virtual embodiments. Second, evidence from comparative neuroscience, convergent evolution, neurotechnology, and philosophy of mind suggests that the mechanisms relevant for consciousness are largely substrate-independent and may therefore be implemented in both biological and artificial systems. Third, although unknown physical mechanisms cannot be excluded, there is currently no empirical evidence for biological processes that are uniquely necessary for consciousness. Taken together, these considerations support the view that consciousness is best understood as an emergent property of recursively embodied, environmentally embedded cognitive systems rather than of biological matter itself. If so, biological brains, artificial neural networks, neuromorphic hardware, and even virtual agents may constitute plausible candidates for future artificial consciousness.

## 1 Introduction

### 1.1 Central question

The question of whether artificial systems can become conscious has moved from speculative philosophy toward an increasingly empirical scientific debate. At the same time, neuroscience and AI research are converging conceptually through shared interests in world models, predictive processing, self-representation, and adaptive behavior.

Recent advances in artificial intelligence and computational neuroscience have enabled increasingly detailed simulations of biological nervous systems. In a striking example, researchers reconstructed the fruit fly connectome and embedded the resulting neural system into a virtual body acting within a simulated environment (Vopson, 2026). Once coupled to this closed sensorimotor loop, the virtual agent began to exhibit complex and partially spontaneous behaviors such as grooming and food-seeking. Such developments naturally raise the question of what may happen as these approaches scale further: from insects to mice, cats, or eventually even human-level neural architectures embedded in artificial or virtual bodies and worlds comparable to a “mind uploading scenario” (Chalmers, 2014, 2022). Would the simulated human be conscious, and could we find out?

Nevertheless, consciousness remains difficult to investigate scientifically because subjective experience, or qualia, is inherently private and cannot be directly observed from a third-person perspective or objectively measured (Chalmers, 1995b). Thus, the existence of consciousness even in other humans, infants, and non-human animals is inferred from converging evidence rather than observed directly (Nagel, 1974; Kouider et al., 2013; Birch et al., 2020).

As emphasized by Thomas Nagel, the existence of subjective experience raises the question of what it is like to be a conscious system (Nagel, 1974). Consequently, purely behavioral criteria such as the Turing Test may be insufficient, since intelligent behavior alone does not necessarily imply phenomenological awareness (Turing, 1950) [see also Searle (1980)].

### 1.2 Central thesis

Here, we argue for three central claims.

1. Consciousness does not emerge from cognition alone, but from a dynamically closed loop between a cognitive system, a body, and an environment (Damasio, 1999; Krauss and Maier, 2020).
2. Consciousness-relevant cognitive organization may be multiply realizable in biological brains, artificial neural systems, neuromorphic hardware, or computational simulations (Putnam, 1980; Lewis, 1972).
3. Neither body nor environment necessarily need to be biological or even physically real, but may in principle also exist as fully virtual or simulated systems.

## 2 Consciousness requires a closed loop

According to Antonio Damasio, consciousness does not emerge from abstract cognition alone, but from the continuous regulation and representation of bodily states (Damasio, 1999). In his framework, the proto-self consists of dynamically changing neural mappings of the organism's internal condition, primarily related to homeostasis and interoception. Core consciousness emerges when the organism continuously integrates these bodily representations with sensory interaction and ongoing behavior. Consciousness is therefore fundamentally grounded in the recursive interaction between brain, body, and environment rather than in isolated information processing alone.

Similarly, Anil Seth conceptualizes perception and selfhood within the framework of predictive processing (Seth, 2014). According to this view, the brain continuously generates predictions about both the external world and the internal bodily state and updates these predictions through sensory feedback. Conscious perception therefore represents a form of “controlled hallucination” emerging from recursive prediction loops. Importantly, Seth emphasizes interoception as a central component of conscious selfhood, suggesting that the experience of being a self emerges from the brain's ongoing predictive regulation of the body (Seth, 2013).

These theories collectively suggest that isolated cognition alone may be insufficient for the emergence of consciousness. A world model cannot emerge without continuous interaction with an environment, and a self-model requires some form of embodiment and internal regulation. Consciousness therefore appears to depend on recursive sensorimotor loops that continuously couple the cognitive system to both body and world (Figure 1). Without such dynamic closure, information processing may remain abstract and disconnected from subjective experience.

**Figure 1:**
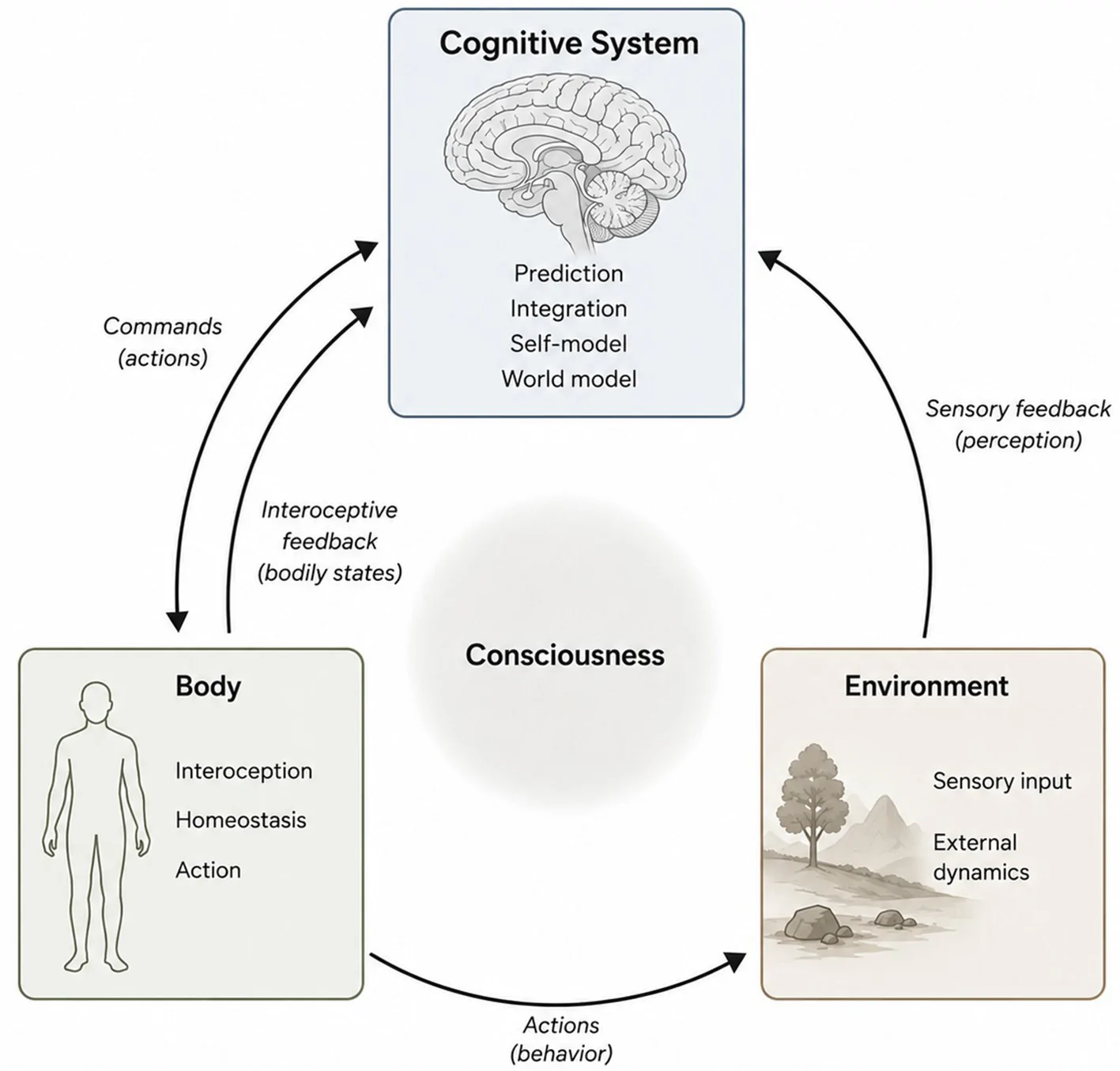
Biological closed-loop model of consciousness. Conceptual framework of consciousness as a dynamically closed interaction loop between a cognitive system, a body, and an environment. The cognitive system continuously integrates sensory input from the environment, interoceptive feedback from the body, prediction, self-modeling, and world-modeling to guide behavior and action. Bodily states and environmental interactions recursively shape ongoing cognitive processing through sensorimotor feedback loops. This biologically grounded version is in line with theories of embodied and predictive consciousness proposed by Damasio (1999) and Seth (2014).

### 2.1 Cross-theoretical integration

Recursive embodied closure is compatible with several influential theories of consciousness, although these theories emphasize different mechanisms and explanatory levels. Within predictive processing accounts, conscious perception depends on continuously updated predictions about external and internal states (Seth, 2013, 2014). Prediction errors generated through sensory and interoceptive feedback recursively modify the system's world model and self-model. The proposed closed-loop framework extends this perspective by treating prediction not as an isolated computational operation, but as part of an ongoing cycle linking perception, bodily regulation, action, and environmental change.

Embodied and enactive approaches place even stronger emphasis on this continuous coupling. From these perspectives, cognition and conscious experience do not arise exclusively from internal representations, but from the active engagement of an organism with its body and environment (Varela et al., 2017; Thompson, 2010). Recursive embodied closure captures this idea by defining the cognitive system, body, and environment as mutually influencing components of a single dynamical process. The body thereby contributes not merely sensory input, but also homeostatic demands, action possibilities, and a stable distinction between self-generated and externally generated changes.

The framework is also compatible with Global Workspace Theory. According to this family of theories, conscious access involves the integration and global availability of information across otherwise specialized cognitive systems (Baars, 1993; Dehaene and Changeux, 2011). A recursively embodied system similarly requires information originating from perception, interoception, memory, prediction, and action control to become available for flexible behavior and coordinated regulation. Recursive embodied closure does not specify the neural or computational architecture through which such global availability is achieved, but instead describes the broader interaction loop within which workspace-like integration may become functionally relevant.

Higher-Order Theories propose that a mental state becomes conscious when the system represents itself as being in that state (Lau and Rosenthal, 2011). Within the present framework, such metarepresentational processes can be understood as components of self-modeling. A system that continuously represents not only the external world, but also its own perceptual, bodily, and cognitive states, may develop increasingly differentiated representations of itself as the subject of experience and action.

Finally, Integrated Information Theory and Recurrent Processing Theory emphasize internally integrated causal organization and recurrent neural dynamics, respectively (Tononi, 2004; Tononi and Koch, 2015; Lamme, 2006). Recursive interactions within the cognitive system and across cognition, body, and environment may support both recurrent processing and complex causal integration. The present framework, however, does not assume that recurrence or integration alone is sufficient for consciousness. Rather, it proposes that these dynamics may acquire consciousness-related significance when they participate in a stable and self-maintaining loop involving prediction, regulation, action, and feedback.

Recursive embodied closure is not intended as a competing theory of consciousness, but as a higher-level organizational framework within which several theory-specific mechanisms may operate. It therefore provides a common descriptive level for comparing theories that differ in their preferred mechanisms, terminology, and explanatory ambitions. At the same time, these theories may specify complementary conditions that a recursively embodied system would need to satisfy before it becomes a plausible candidate for conscious experience.

## 3 Multiple realizability of consciousness-relevant organization

### 3.1 Evolutionary evidence against biological exclusivity

Evolutionary biology provides strong evidence against the assumption that consciousness or advanced cognition depend on a single specific biological architecture. Complex cognitive abilities have evolved independently in mammals, birds, and cephalopods despite fundamentally different nervous system organizations (Birch et al., 2020). In addition, recent work on distributed intelligence in slime molds, fungal networks, and bioelectric signaling systems, including research by Michael Levin, suggests that adaptive and goal-directed behavior may emerge in a wide range of biological substrates (Levin, 2022). These observations suggest that similar cognitive properties can emerge in very different biological systems. However, one crucial question remains unresolved: Does consciousness really require life, and if so, which properties of living organization are relevant to conscious experience? Indeed, Anil Seth argues that only living things can develop a consciousness (Seth, 2025, 2026). However, this raises two key follow-up questions: Which properties of life actually play a role for conscious experiences?, and How should life itself be defined (Mariscal and Doolittle, 2020; Bedau, 1996; Benner, 2010; Seth, 2013)?. Recent work by Bender and colleagues further complicates any sharp distinction between living and non-living systems. Using large language models to compare definitions of life proposed by experts from different disciplines, they found not a small number of clearly separated conceptual categories, but a continuous landscape of overlapping views within a common latent space (Bender et al., 2025). This suggests that “life” itself may be better understood as a multidimensional continuum rather than as a binary property with a universally agreed boundary. If so, grounding consciousness exclusively in life does not by itself resolve the substrate question, but shifts it to the identification of those dimensions of living organization that are actually relevant for conscious experience. What does this point of view mean for hypothetical silicon-based alien life forms, synthetic organisms, or artificial systems that instantiate some, but not all, of the properties conventionally associated with life?

These arguments therefore justify considering whether consciousness could also emerge in non-biological or, from a biological perspective, even “non-living” physical systems such as neuromorphic hardware.

### 3.2 Ship of theseus and gradual replacement

A central argument supporting this possibility is the philosophical Ship of Theseus thought experiment applied to the brain (Scaltsas, 1980). If biological neurons were gradually replaced by functionally equivalent artificial hardware components, at which exact point should consciousness disappear (Chalmers, 1995a)? Arguments such as fading qualia and absent qualia suggest that a sudden loss of subjective experience despite preserved functionality, behavior, memory, and self-modeling would appear highly implausible. These considerations challenge strict biological essentialism and support the possibility that consciousness depends primarily on functional and dynamical organization rather than exclusively on biological tissue (Putnam, 1980) (Figure 2).

**Figure 2:**
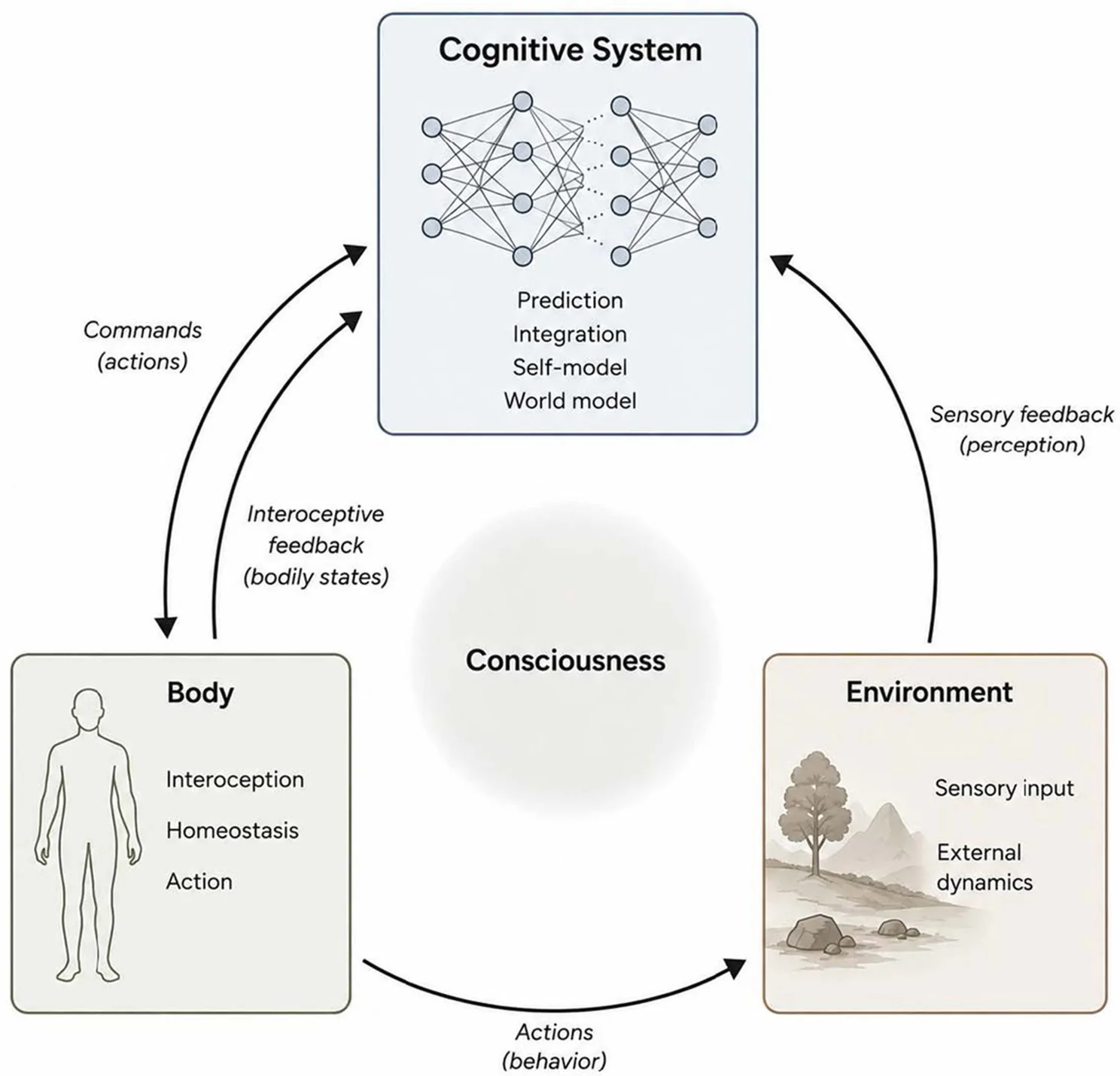
Substrate-independent cognitive system embedded in biological embodiment and real world. Conceptual illustration of the hypothesis that consciousness-relevant cognitive processes may be largely substrate-independent. Comparative neuroscience and convergent evolution demonstrate that complex cognition has emerged across fundamentally different biological architectures, including mammals, birds, and cephalopods, suggesting that conscious processing may depend more on functional and dynamical organization than on a specific biological implementation. Consequently, similar cognitive principles may in principle also be realizable in non-biological physical substrates, such as neuromorphic hardware, hypothetical silicon-based life forms, or even as entirely virtual software agents, while consciousness itself remains grounded in recursively closed interactions between cognition, body, and environment.

Current developments in brain-computer interfaces and neural prosthetics further support this perspective. Artificial sensory implants and motor prostheses already integrate synthetic hardware components into neural processing without any indication that subjective experience is disrupted (Bashford and Mehring, 2016; Gallagher, 2006). If consciousness tolerates partial replacement and hybridization between biological and artificial systems, it becomes increasingly difficult to argue that biological matter itself is uniquely necessary for conscious experience. This naturally raises the next question: if the relevant cognitive components do not need to be biological, do they need to be physically realized at all, or could they also exist as fully simulated systems? Importantly, these two levels of realization (physical implementation and purely virtual simulation) are often conflated in current debates, although they represent conceptually distinct questions.

## 4 Body and world need not be physically real

So far, we argued that the cognitive system itself may be substrate-independent and therefore not necessarily tied to biological tissue. The next question is whether body and environment must remain physically real once a conscious biological cognitive system is assumed. In the following, we argue that even for a biological brain, both embodiment and environmental interaction could in principle be fully virtual or simulated while still maintaining the closed-loop dynamics required for conscious experience.

### 4.1 Virtual embodiment

From this perspective, the crucial factor may not be biological embodiment itself, but the existence of stable closed-loop dynamics between cognition, body, and environment. In principle, a virtual body could provide many of the same functional properties associated with conscious systems, including interoception, agency, self-other distinction, homeostatic regulation, and prediction error minimization. If consciousness emerges from recursive embodied interaction rather than from specific biological matter, then virtual embodiment may be sufficient to support conscious experience (Figure 3).

**Figure 3:**
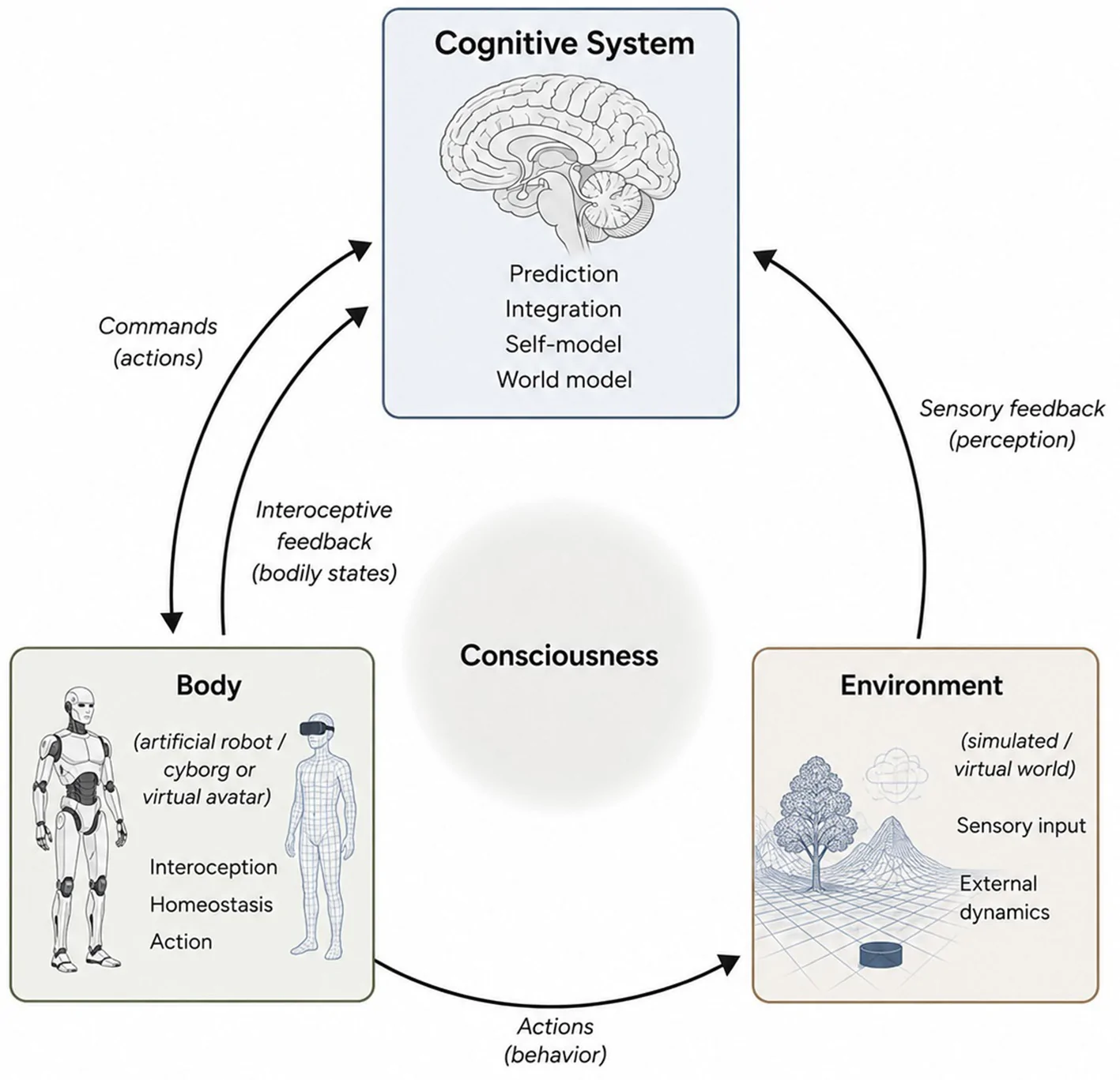
Virtual embodiment and simulated environments in conscious systems. Conceptual illustration of the hypothesis that neither embodiment nor environmental interaction necessarily need to be biological or physically real for consciousness-relevant dynamics to emerge. If conscious experience depends primarily on recursively closed sensorimotor and predictive interaction loops, then artificial cyborg or even completely virtual bodies and simulated environments (virtual reality) may in principle provide the functional conditions required for self-modeling, agency, interoception, and world-modeling. From this perspective, coherent interaction dynamics rather than physical materiality itself may constitute the crucial foundation of conscious experience. At its core, this idea corresponds to the classical “brain in the vat” thought experiment, according to which a (biological) cognitive system connected to sufficiently coherent simulated sensorimotor inputs may be unable to distinguish virtual reality from physical reality itself.

### 4.2 Brain in the vat

The classical brain-in-a-vat thought experiment further extends this argument (Putnam, 1981; Smith, 1984; Wright, 1992). If a biological cognitive system were connected to perfectly simulated sensorimotor inputs, it is unclear whether the system could distinguish such a simulation from physical reality itself. From a phenomenological perspective, a sufficiently coherent virtual environment may therefore become indistinguishable from a physically real one. This suggests that conscious experience may depend less on the material reality of the environment and more on the consistency and recursive structure of the underlying interaction loops. Note that, the brain-in-a-vat thought experiment concerns the source of sensory input for an already conscious biological system and does not by itself establish consciousness in a simulation. Thus, it is questionable whether this though experiment proves that a physical body is not necessary for the continuation of conscious experience.

### 4.3 Simulated worlds can be semantically meaningful

This perspective also challenges arguments against simulated cognition based on missing semantic grounding, such as those proposed by Putnam (1981). Meaning may emerge not from direct contact with physical reality itself, but from stable interaction dynamics within an environment. Even purely virtual environments, including computer games and online social worlds, can acquire substantial meaning and emotional relevance for human agents (Suchan and Bhatt, 2017; Qi et al., 2026). Consequently, simulated environments may in principle also provide sufficient semantic structure for artificial cognitive systems.

## 5 From virtual embodiment to fully simulated cognitive systems

Taken together, the previous arguments raise the possibility that not only body and environment, but also the cognitive system itself could in principle be simulated. If consciousness depends primarily on recursively closed interaction loops between cognition, embodiment, and world, then the relevant factor may be the organizational structure of the system rather than the physical nature of its implementation.

### 5.1 Fruit fly connectome simulations

Recent fruit fly connectome simulations illustrate this development particularly vividly (Vopson, 2026). In these experiments, simulated neural circuits were connected to virtual bodies acting within simulated environments, thereby forming complete sensorimotor interaction loops. Once embedded into such virtual worlds, the agents began to display increasingly adaptive and seemingly spontaneous behaviors, including grooming, locomotion, and food-seeking (Figure 4). Although these systems remain far from biological organisms in complexity, they raise an important question: if increasingly realistic simulated brains embedded in virtual bodies and environments continue to scale toward higher levels of complexity, at what point does the distinction between mere simulation and genuine cognition become conceptually unclear? Or in other words: Is a simulated entity aware of its existence?

**Figure 4:**
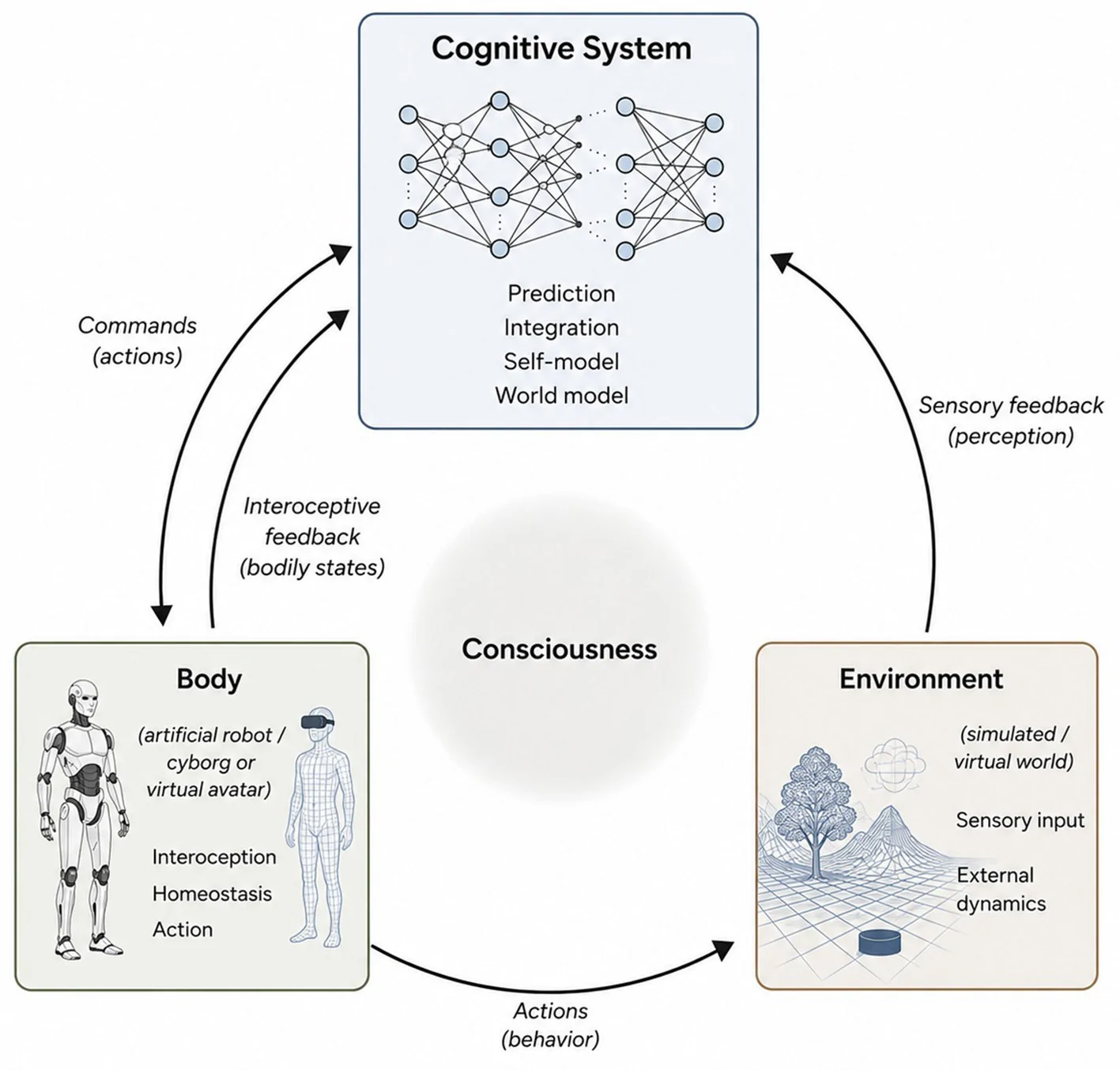
Simulated connectomes, artificial or virtual organisms and virtual worlds. Conceptual illustration of connectome-based simulations of biological nervous systems embedded in closed sensorimotor interaction loops. Recent advances in computational neuroscience have enabled increasingly realistic simulations of biological nervous systems, including virtual fruit fly connectomes coupled to artificial bodies within simulated environments. Once embedded into such recursively closed loops between neural processing, embodiment, and environmental interaction, these systems can exhibit adaptive and partially spontaneous behaviors such as locomotion, grooming, and food-seeking. These developments raise the fundamental question of whether sufficiently detailed simulated nervous systems may eventually not only reproduce behavior, but also instantiate elementary forms of conscious processing.

### 5.2 AI systems and emerging world models

Recent advances in artificial intelligence further support this perspective. Large language models, reinforcement learning agents, and embodied AI systems increasingly develop complex internal representations of both environment and agent-related states (Immertreu et al., 2025; Krauss and Maier, 2020; Krauss, 2024). In particular, world models, self-representation, and predictive internal dynamics emerge spontaneously in sufficiently complex learning systems. While this does not imply that current AI systems are conscious, it suggests that some computational pre-requisites traditionally associated with cognition and selfhood may arise naturally from adaptive interaction with complex environments. Social environments may be particularly important because they require several individuals to model and respond to one another. This idea is supported by the fact that brain size is highly correlated to the actual number of social contacts (Dunbar, 1992, 1996, 2004). For humans the maximum number of stable contacts is approximately 150, the so-called “Dunbar Number” (De Ruiter et al., 2011).

## 6 Empirical predictions

The framework of recursive embodied closure generates several empirically testable predictions. First, artificial systems embedded in persistent sensorimotor and homeostatic feedback loops should develop more stable and context-sensitive self-models than otherwise comparable systems trained without such closed-loop interaction. This could be assessed through measures of self–other distinction, metacognitive consistency, adaptation to bodily perturbations, and the temporal stability of internal self-representations.

Second, experimentally perturbing artificial interoceptive or homeostatic variables should systematically influence attention, decision-making, action selection, self-representation, and behavioral reports. If such internal variables are constitutive components of the system's self-model rather than merely auxiliary control signals, their manipulation should produce coordinated changes across several functional domains.

Third, physical and virtual embodiments should give rise to comparable consciousness-related organizational and dynamical markers when their causal interaction structures are functionally equivalent. Differences between robotic and simulated agents should therefore depend less on whether the body and environment are physically real than on the richness, persistence, and causal coherence of the corresponding interaction loops.

Fourth, gradual replacement of biological components by functionally and dynamically equivalent artificial components should not produce abrupt discontinuities in consciousness-related markers. Instead, measures of integration, recurrent processing, self-model stability, adaptive regulation, and metacognitive access should change continuously unless a functionally critical component of the closed loop is disrupted.

Finally, selective disruption of components of the recursive loop should produce dissociable and potentially time-dependent effects on consciousness-related functions. Loss of overt motor output alone does not abolish consciousness, as illustrated by patients with locked-in syndrome, in whom conscious awareness and personal identity may remain preserved despite profound paralysis. However, prolonged loss of action, communication, and external temporal cues may weaken agency, alter sleep–wake organization, and reduce the continuous updating of bodily and self-related models, particularly in the complete locked-in state. More profound disturbances should occur when motor disconnection is accompanied by impaired integration of exteroceptive, interoceptive, and internally recurrent signals. The framework therefore predicts that consciousness-related impairments depend not simply on the absence of observable action, but on the extent, duration, and anatomical level of disruption within the cognition–body–environment loop.

These predictions do not provide a direct measure of phenomenal experience. They specify observable consequences that would be expected if recursive embodied closure constitutes a necessary organizational condition for consciousness. The framework could therefore be challenged if stable self-modeling, metacognitive access, and consciousness-related dynamics emerged independently of persistent embodied interaction, or if substantial disruption of the proposed closed loop had no systematic functional or dynamical consequences.

## 7 Discussion and conclusion

### 7.1 Summary

Recursive embodied closure describes consciousness as arising from the continuous interaction between cognition, bodily processes, and the environment. According to this framework, conscious processing cannot be understood by considering cognition in isolation. A cognitive system continuously receives information about both the external world and its own internal state, uses this information to build and update models of the world and of itself, generates predictions, selects actions, and evaluates the consequences of these actions through new sensory and bodily feedback. Perception, internal regulation, prediction, action, and environmental interaction therefore form a continuous recursive process in which each part influences the others. The central hypothesis of our framework is that the organization of this loop may be more important for consciousness than the specific biological material from which its components are built. If this is correct, similar organizational principles could in principle be realized in artificial systems. The cognitive system could be implemented in artificial hardware, while the body and environment could be biological, artificial, or entirely virtual. Even a fully simulated agent could therefore, in principle, realize the relevant closed loop organization if cognition, internal regulation, embodiment, action, and environmental feedback remain continuously and causally connected.

Importantly, recursive embodied closure does not assume the existence of a separate central “pilot”, inner observer, or homunculus that receives information from the rest of the system and decides how the system should act. Such an assumption would not explain consciousness, because it would simply shift the problem to another unexplained entity within the system, corresponding to the classical homunculus problem (Lorincz and Szirtes, 2003; Metzinger, 2007, 2010; Safron, 2021; Crick and Koch, 2000). Instead, the subject is understood as a persistent and continuously updated self model that emerges from the integration of bodily states, perception, memory, prediction, agency, and metacognitive processes. This self model is not a separate observer of the system, but part of the same ongoing dynamics through which the system represents and regulates itself while interacting with its environment. Consciousness is therefore not located in a single internal component or control center, but is proposed to arise from the coordinated dynamics of the recursively closed system as a whole.

### 7.2 Related work and counterarguments

#### 7.2.1 Why consciousness may not be like rain or gravity

A common objection against artificial consciousness argues that simulations do not reproduce the physical properties of the systems they model. Simulated weather does not generate actual rain, simulated black holes do not produce real gravity, and simulated digestion does not digest physical food (Tononi and Koch, 2015; Koch, 2021). By analogy, critics argue that a simulated mind may imitate cognition and behavior without generating genuine conscious experience.

At first glance, the objection appears compelling. After all, computer simulations of physical processes do not normally instantiate the physical phenomena they represent. A simulation of rainfall does not make objects wet, a simulation of a black hole does not generate gravity, and a simulation of digestion does not metabolize food. If consciousness were analogous to such physical processes, it would seem reasonable to conclude that a simulated mind could at best imitate conscious behavior without generating genuine subjective experience.

However, consciousness may differ in important respects from phenomena such as rain or gravity. Weather and gravitation are externally observable physical processes with directly measurable effects, whereas consciousness is accessible only from a first-person perspective as subjective experience. Consequently, the analogy does not settle the question, because consciousness may depend on internally realized organizational and dynamical states rather than on the externally observable physical properties that define rain or gravity (Nagel, 1974).

An even stronger version of this objection is that conscious experience may depend on currently unknown biological or physical mechanisms that cannot be fully captured by functional or computational organization alone (Tononi and Koch, 2015).

Thus, living nervous systems indeed involve autopoiesis, metabolism, bioelectricity, thermodynamic processes, and subcellular organization (Miller et al., 2025; Levin, 2022). These mechanisms may be important, but none has yet been specified as uniquely necessary for consciousness.

Nevertheless, the possibility that one of these mechanisms is a fundamental pre-requesite for consciousness cannot be excluded in principle. Consequently, our current scientific understanding of consciousness remains incomplete.

However, as long as such mechanisms remain unspecified, the claim that consciousness is exclusively tied to biological matter remains difficult to evaluate empirically. Any sufficiently advanced artificial system could otherwise be denied consciousness by appealing to hypothetical hidden properties of biological tissue that have not yet been independently identified or characterized.

From a scientific perspective, explanatory frameworks should avoid introducing additional assumptions unless they are required by the available evidence. Following this heuristic principle, often associated with Occam's razor (Thorburn, 1918), it may therefore be preferable to first investigate whether known organizational, dynamical, and computational principles are sufficient to account for consciousness before postulating additional undiscovered mechanisms. This does not imply that such mechanisms are impossible; rather, it reflects a preference for parsimonious explanations until further empirical evidence becomes available.

Moreover, even if consciousness were ultimately linked to currently unknown physical fields or processes, this would not necessarily exclude artificial consciousness altogether. Such mechanisms could in principle be reproduced within sufficiently advanced artificial systems or neuromorphic hardware, analogous to how electromagnetic phenomena can be generated and controlled through technological means. In that case, the debate would shift from whether artificial consciousness is possible to which physical conditions are necessary for its realization.

Consequently, the existence of currently unknown biological mechanisms, while possible, does not by itself constitute an argument against artificial consciousness. Instead, it highlights an important open empirical question regarding the physical foundations of conscious experience (Tononi and Koch, 2015). Until such mechanisms are identified, theories emphasizing recursive interactions between cognition, embodiment, and environment remain viable candidates for explaining consciousness in both biological and artificial systems.

Nevertheless, it has to be considered that also connectome simulations depend on choices about neural dynamics, environmental cues, biomechanics, and evaluation criteria, and expectancy effects may influence their interpretation (Rosenthal and Rubin, 1978), which also introduce additional arbitrary parameters contradicting Occam's razor principle (Thorburn, 1918). Sensitivity analyses, comparison models, blinded evaluation, and out-of-distribution tests should therefore accompany claims based on emergent behavior.

#### 7.2.2 Non-computability, biological specificity, and functional equivalence

One important objection to artificial consciousness is that consciousness may not be fully explainable in computational terms. Several authors have argued that life or conscious experience may involve properties that cannot be completely described by algorithms or formal models (Penrose, 1994; Song, 2007; Marshall, 2021; Kauffman and Roli, 2023; Kak, 2024; Garte et al., 2025; Miller et al., 2025). If this is correct, an artificial system could reproduce complex behavior, internal representations, or even a detailed self model without necessarily having subjective experience.

A related concern is that some biological processes may play a special role in consciousness. In living organisms, interoception, homeostatic regulation, and metabolism are real physiological processes that continuously influence the state of the organism (Damasio, 1999; Seth, 2013; Singer and Damasio, 2025). A simulated system can represent similar internal states and reproduce some of their functions, but these simulated variables are not physically identical to the biological processes. It therefore remains possible that some properties of biological regulation are important for conscious experience and are not captured by a purely computational simulation.

However, this physical difference alone does not show that biology is necessary for consciousness. A simulated process can be physically different from a biological process while still performing a similar function. This distinction between physical realization and functional organization is also relevant to theories of extended cognition. The extended mind thesis proposes that cognitive processes can include components outside the biological brain when these components are continuously and reciprocally connected to the agent (Clark and Chalmers, 1998). This suggests that at least some cognitive functions do not have to be located entirely within biological nervous tissue.

Importantly, however, extending cognition beyond the biological brain does not automatically imply an extension of consciousness (Rapoport, 2012). In the same way, individual functional signs should not be treated as direct evidence of subjective experience. For example, an artificial system may report uncertainty, estimate its own confidence, or update an internal model without necessarily experiencing doubt or confidence. Such observations become more informative when they appear together with broader properties such as a persistent self model, regulation of internal states, recurrent integration of information, autonomous action, and stable adaptation to unexpected changes. Even then, these properties should be understood as possible indicators of consciousness rather than as direct proof of subjective experience.

Taken together, these arguments show why both sides of the debate should be treated with caution. The fact that no uniquely biological mechanism for consciousness has been identified does not prove that computation alone is sufficient. At the same time, the fact that all systems we currently accept as conscious are biological does not prove that biological matter is necessary for consciousness.

Importantly, the boundaries of what we accept as conscious have changed several times in the past. Non-human animals were once often regarded as largely automatic and without conscious experience, whereas today consciousness is considered plausible in many different animal species (Birch et al., 2020; Irwin, 2020). A similar change can be seen in infants. Because infants cannot verbally report their experiences, their consciousness was long difficult to assess, whereas more recent evidence supports conscious processing much earlier in development than some previous theories assumed (Bayne et al., 2023; Kouider et al., 2013).

These examples should be understood as a warning. The boundaries of consciousness have been pushed before, and they may be pushed again as new theories, methods, and evidence become available. This does not mean that artificial systems are conscious. It means that we should be careful not to define the limits of consciousness only on the basis of the systems we currently accept as conscious. The key open question is therefore whether consciousness mainly depends on the functional and dynamical organization of a system, or whether specific biological properties are fundamentally required. Answering this question will require clear criteria and empirical predictions that can distinguish between these possibilities.

### 7.3 Conclusion and outlook

Taken together, the presented arguments suggest that consciousness may not be a property uniquely tied to biological matter, but rather an emergent phenomenon arising from recursively embodied and environmentally embedded information processing systems. Current theories of consciousness increasingly emphasize the importance of dynamically closed interaction loops between cognition, body, and environment instead of isolated computation alone. At the same time, evidence from evolutionary biology, neuroscience, artificial intelligence, and neurotechnology suggests that the cognitive system itself may be largely substrate-independent.

If consciousness depends on the recursive organization of the loop rather than on the specific biological implementation of its components, then gradual replacement of cognition, body, and environment should in principle preserve conscious processing.

More generally, research in dynamical systems and complex systems theory demonstrates that many emergent phenomena can be reproduced independently of their specific physical realization. Conway's Game of Life, for example, generates identical emergent dynamics whether implemented on a computer or manually simulated using pencil and paper (Johnston and Greene, 2022; Rendell, 2011). Similarly, flocking behavior in birds can be accurately reproduced in computational simulations through simple interaction rules. These examples illustrate that complex and emergent system dynamics are often determined primarily by organizational principles and interaction structures rather than by the specific material substrate in which they are realized. Consciousness is probably more similar to these emergent phenomena than to actual physical forces and fields or phenomena such as gravity, electromagnetism, or rain.

If this view is correct, neither the body nor the environment necessarily need to be biological or even physically real, but may in principle also exist as virtual or simulated systems. Within the framework of the Platonic Representation Hypothesis, similar representational and cognitive structures may emerge across different substrates whenever sufficiently similar organizational principles are realized (Gröger et al., 2026; Li and Walsh, 2026).

Consequently, biological brains, artificial neural systems, neuromorphic hardware, and potentially even fully virtual agents may converge toward functionally and phenomenologically similar states.

Although the possibility remains that consciousness depends on currently unknown physical mechanisms, this assumption would ultimately require empirical specification. Until such evidence exists, increasingly sophisticated artificial agents embedded in coherent sensorimotor interaction loops may represent plausible candidates for future forms of artificial consciousness.

Thus, it is important to consider the possibility of simulated consciousness and to avoid defining biological life as the basis of consciousness simply to avoid the fourth or fifth scientific affront to humanity (Freud, 1917; Floridi, 2014; Krauss, 2024).

Taken together, the arguments presented here suggest that a simulated animal (or human), or any other kind of virtual agent embedded in a coherent sensorimotor loop, may in principle become aware of its own existence (Figure 5). If and only if the assumptions made in this paper are correct, the simulated fruit fly might represent one of the simplest currently available candidate systems for investigating the emergence of minimal consciousness. Although the existence of digital consciousness cannot be proven at this point, it cannot be ruled out with certainty either.

**Figure 5:**
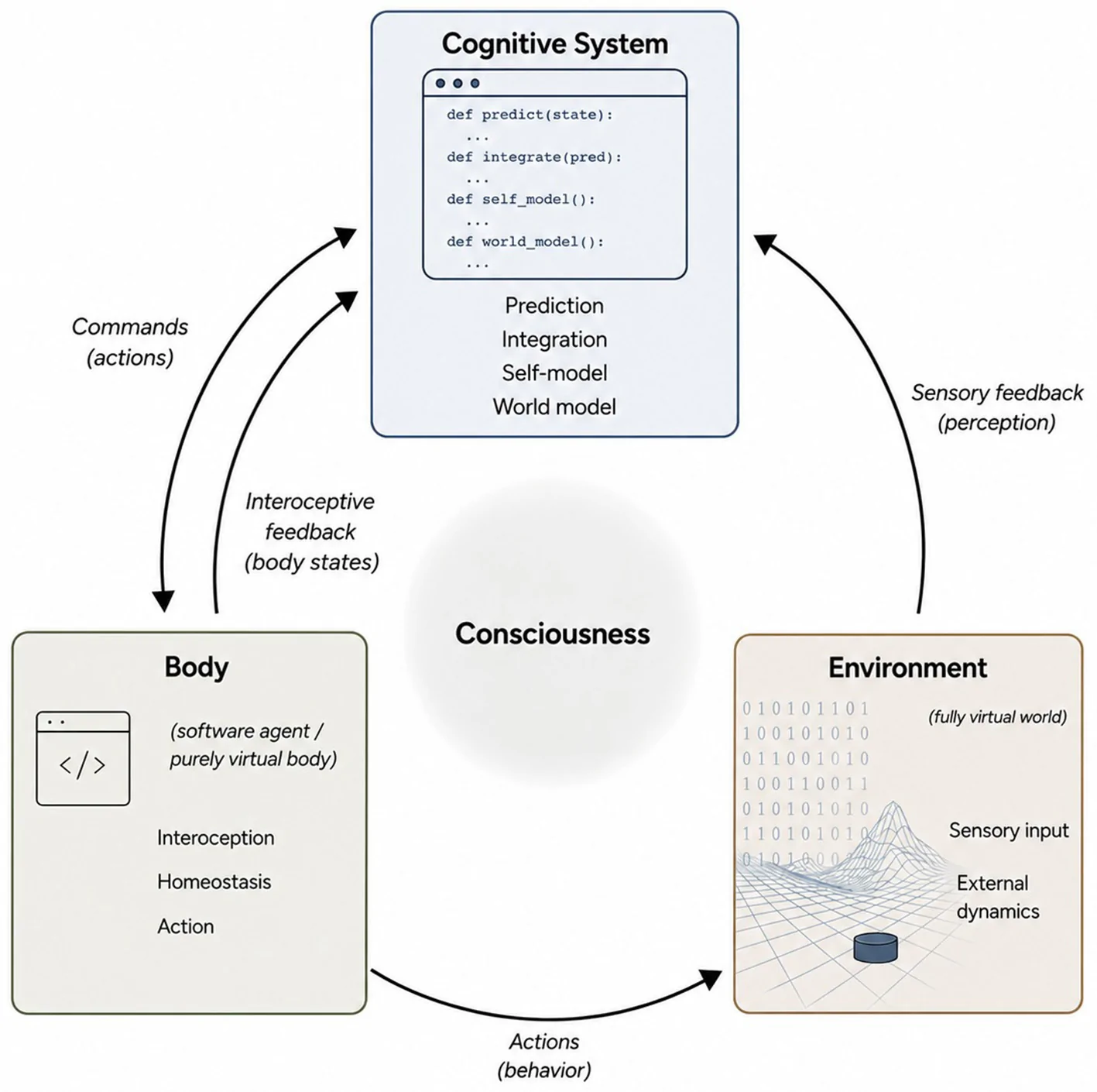
Fully virtual cognitive agents in simulated digital worlds. Conceptual illustration of fully virtual cognitive systems embedded in simulated environments and interacting through recursively closed digital sensorimotor loops. In such systems, cognition, embodiment, and environmental interaction are realized entirely within computational substrates, allowing artificial agents to develop world models, self-representations, adaptive behavior, and social interaction inside coherent virtual worlds. Recent advances in artificial intelligence, reinforcement learning, and agentic AI suggest that increasingly complex internal representations and predictive dynamics can emerge naturally from closed-loop interaction with sufficiently rich environments. Within this framework, consciousness-related organizational principles may in principle arise independently of biological realization, provided that sufficiently stable and recursively embodied interaction dynamics are maintained.

## Data Availability

The original contributions presented in the study are included in the article/supplementary material, further inquiries can be directed to the corresponding author.
